# Supplementary material for: A tRNA half modulates translation as stress response in Trypanosoma brucei
Source: Nat Commun. 2019 Jan 10;10:118. doi: 10.1038/s41467-018-07949-6 (PMC6328589; doi:10.1038/s41467-018-07949-6)
Supplement: Supplementary file 1 — Supplementary Information [file 41467_2018_7949_MOESM1_ESM.pdf]

Supplementary Information

A tRNA half modulates translation as stress response in *Trypanosoma brucei*

Roger Fricker[1,2,#], Rebecca Brogli[1,2,#], Hannes Luidalepp[1], Leander Wyss[1,2], Michel Fasnacht[1,2], Oliver Joss[1], Marek Zywicki[3], Mark Helm[4], André Schneider[1], Marina Cristodero[1]* and Norbert Polacek[1]*

[1]Department of Chemistry and Biochemistry, University of Bern, Freiestrasse 3, 3012 Bern, Switzerland
[2]Graduate School for Cellular and Biomedical Sciences, University of Bern, Bern, Switzerland
[3]Department of Computational Biology, Institute of Molecular Biology and Biotechnology, Adam Mickiewicz University, Umultowska 89, 61-614 Poznan, Poland
[4]Institute of Pharmacy and Biochemistry, Johannes Gutenberg-University of Mainz, Staudingerweg 5, D-55128 Mainz, Germany.

[#]equal contribution

*To whom correspondence should be addressed.

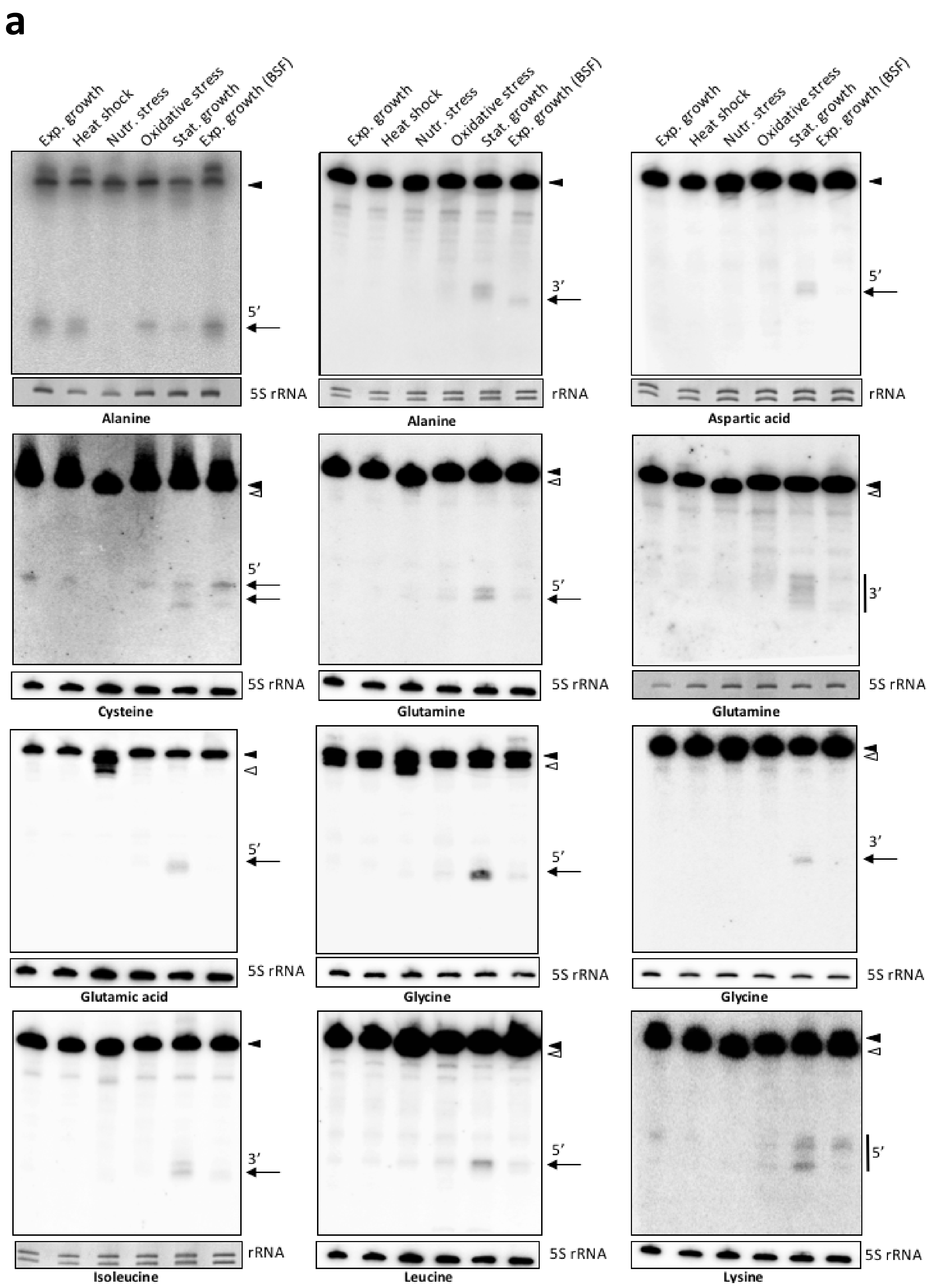
a
Exp. growth
Heat shock
Nutr. stress
Oxidative stress
Stat. growth
Exp. growth (BSF)
5'
3'
5S rRNA
rRNA
Alanine
Alanine
Aspartic acid
Cysteine
Glutamine
Glutamine
Glutamic acid
Glycine
Glycine
Isoleucine
Leucine
Lysine

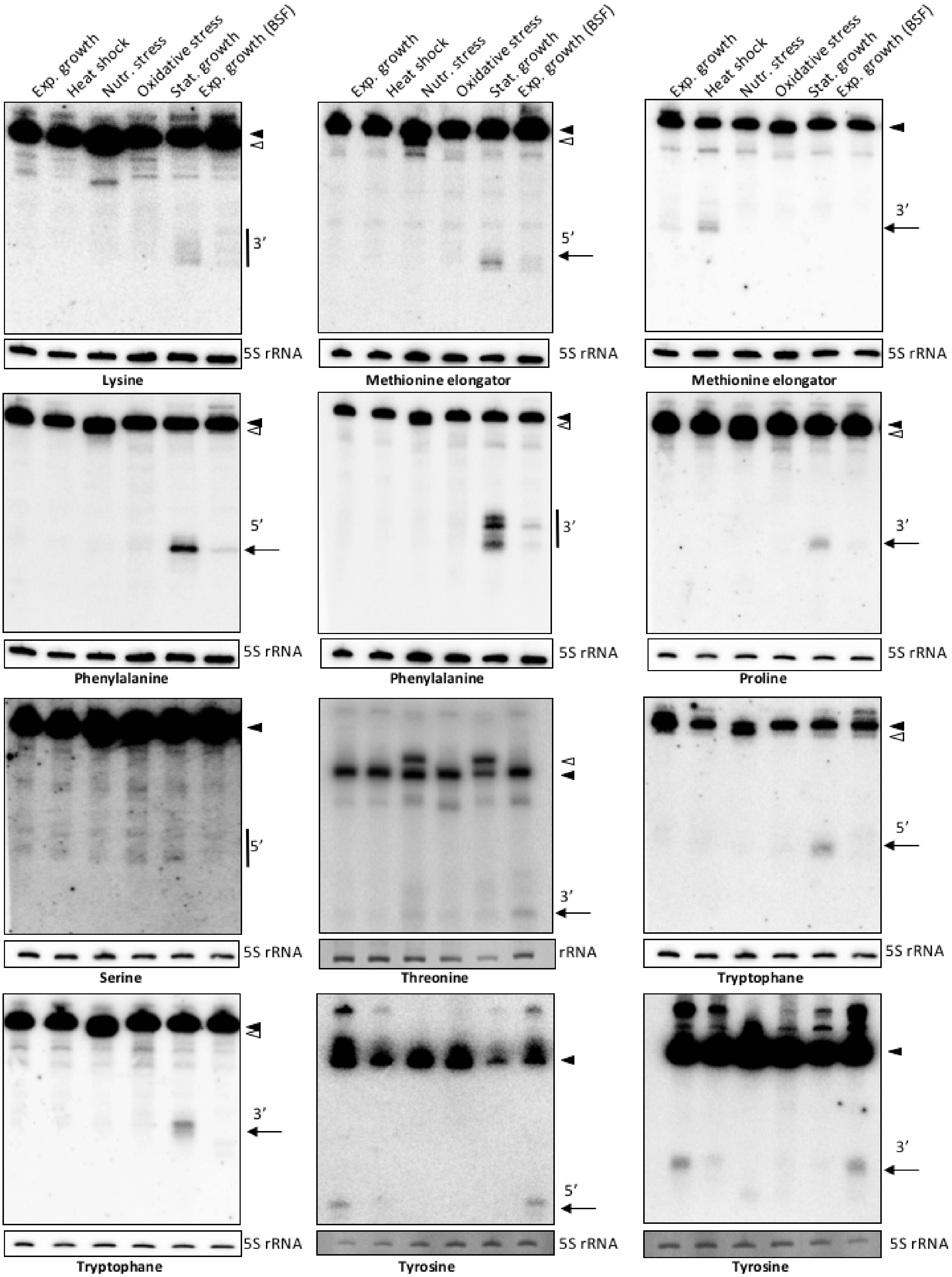
Exp. growth
Heat shock
Nutr. stress
Oxidative stress
Stat. growth
Exp. growth (BSF)
3'
5'
5S rRNA
rRNA
Lysine
Methionine elongator
Methionine elongator
Phenylalanine
Phenylalanine
Proline
Serine
Threonine
Tryptophane
Tryptophane
Tyrosine
Tyrosine

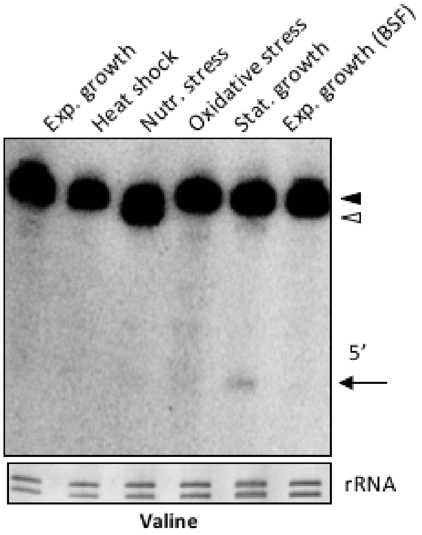
Exp. growth
Heat shock
Nutr. stress
Oxidative stress
Stat. growth
Exp. growth (BSF)
5’
rRNA
Valine

b

		Exp. growth	Heat shock	Nutritional stress	Oxidative stress	Stationary phase	Exp. growth (BSF)	Max. cleavage efficiency
Ala	5'	2.5 ± 0.9	2.5 ± 0.4	*(1.0)*	1.2 ± 0.3	1.0 ± 0.9	**3.0 ± 0.4**	4.4 ± 1.5
	3'	*(1.0)*	1.1 ± 0.1	1.3 ± 0.3	1.6 ± 0.8	**2.9 ± 0.9**	1.7 ± 0.9	3.2 ± 1.7
Arg	5'							
	3'							
Asn	5'							
	3'							
Asp	5'	*(1.0)*	0.7 ± 0.0	0.8 ± 0.1	0.9 ± 0.1	**1.3 ± 0.3**	0.5 ± 0.1	3.4 ± 1.3
	3'							
Cys	5'	3.3 ± 0.4	3.0 ± 1.2	*(1.0)*	3.3 ± 1.5	3.3 ± 1.9	**3.6 ± 0.8**	4.3 ± 0.5
	3'							
Gln	5'	*(1.0)*	1.2 ± 0.6	2.2 ± 1.6	2.6 ± 1.2	**8.3 ± 5.0**	3.4 ± 1.8	1.7 ± 0.0
	3'	*(1.0)*	1.4 ± 0.6	1.7 ± 0.1	2.7 ± 0.1	**8.7 ± 0.9**	2.6 ± 0.9	3.8 ± 0.4
Glu	5'	*(1.0)*	1.7 ± 0.3	3.2 ± 0.8	3.9 ± 0.4	**24.4 ± 1.1**	5.2 ± 1.5	7.6 ± 0.2
	3'							
Gly	5'	*(1.0)*	1.3 ± 0.1	1.2 ± 0.1	3.0 ± 0.3	**20.5 ± 2.2**	3.6 ± 0.2	10.2 ± 1.2
	3'	*(1.0)*	3.4 ± 2.2	3.5 ± 2.3	4.0 ± 2.1	**15.6 ± 10.8**	6.7 ± 4.3	2.1 ± 0.2
His	5'							
	3'							
Ile	5'							
	3'	*(1.0)*	1.0 ± 0.4	2.6 ± 0.7	2.0 ± 0.1	**9.6 ± 2.7**	3.7 ± 0.1	6.0 ± 2.2
Leu	5'	*(1.0)*	1.8 ± 0.4	2.3 ± 0.2	3.8 ± 0.4	**10.8 ± 0.1**	4.1 ± 1.3	6.2 ± 0.1
	3'							
Lys	5'	3.2 ± 1.9	2.4 ± 1.5	*(1.0)*	1.8 ± 0.3	2.0 ± 0.5	**5.3 ± 3.5**	4.3 ± 0.8
	3'	*(1.0)*	0.8 ± 0.3	2.3 ± 0.3	3.6 ± 0.1	**10.0 ± 0.3**	5.1 ± 1.0	2.5 ± 0.1
Met e	5'	*(1.0)*	1.1 ± 0.5	1.0 ± 0.7	1.0 ± 0.6	**7.7 ± 5.5**	3.5 ± 1.9	0.7 ± 0.0
	3'	*(1.0)*	**5.2 ± 0.4**	0.5 ± 0.4	0.3 ± 0.1	0.3 ± 0.2	0.1 ± 0.0	8.9 ± 1.9
Met i	5'							
	3'							
Phe	5'	*(1.0)*	1.4 ± 0.5	1.6 ± 0.4	2.2 ± 0.2	**17.5 ± 1.3**	2.7 ± 1.0	12.2 ± 3.2
	3'	*(1.0)*	0.9 ± 0.0	0.9 ± 0.1	1.1 ± 0.2	**10.1 ± 3.4**	1.6 ± 0.3	34.0 ± 9.9
Pro	5'							
	3'	*(1.0)*	1.2 ± 0.1	1.6 ± 0.3	1.6 ± 0.1	**8.3 ± 2.2**	2.0 ± 0.2	3 ± 0.4
Sel	5'							
	3'							
Ser	5'	*(1.0)*	2.7 ± 1.6	2.2 ± 1.3	4.3 ± 1.6	**6.2 ± 2.6**	6.0 ± 3.5	2.3 ± 0.3
	3'							
Thr	5'							
	3'	*(1.0)*	1.4 ± 0.2	4.4 ± 1.6	3.4 ± 1.9	**18.0 ± 11.1**	11.7 ± 3.7	7.0 ± 0.8
Trp	5'	3.2 ± 2.1	2.8 ± 2.5	*(1.0)*	2.6 ± 0.6	**13.8 ± 8.2**	3.1 ± 2.0	13.5 ± 2.9
	3'	*(1.0)*	2.5 ± 1.6	2.1 ± 1.2	2.7 ± 1.1	**10.4 ± 7.0**	2.4 ± 2.2	3.8 ± 2.7
Tyr	5'	**5.3 ± 2.6**	*(1.0)*	1.7 ± 0.1	2.8 ± 1.7	2.0 ± 1.8	2.6 ± 0.1	4.7 ± 2.2
	3'	2.3 ± 1.0	2.0 ± 0.9	*(1.0)*	1.9 ± 1.1	**4.0 ± 1.7**	3.5 ± 0.2	6.4 ± 1.9
Val	5'	*(1.0)*	2.4 ± 0.6	4.7 ± 2.3	**6.3 ± 3.5**	5.3 ± 3.8	4.4 ± 1.6	1.7 ± 1.4
	3'							

Supplementary Figure 1: Northern blot analyses of tRNA halves on total RNA from *T. brucei*. **a** RNA was isolated from procyclic *T. brucei* cells either during the exponential phase (Exp. growth), or after heat shock, nutritional deprivation (starvation), oxidative stress (H_2O_2), during the stationary phase or from exponentially growing bloodstream form (Exp. growth_BSF). The abundance of tRNA halves (5’ or 3’) and fragments was monitored by northern blot analyses. Full length tRNAs and potentially 3’ trimmed tRNAs are indicated with filled and open arrowheads, respectively. rRNA (5S, rRNA) served as loading control. **b** Quantification of the abundance pattern is summarized for all 5’ and 3’ tRNA halves and tRNA-derived fragments under different conditions. For the quantification of the relative abundance of each fragment, the northern blot signal of exponentially growing procyclic cells was taken as 1.0 and compared to the respective signals under different growth conditions. In a few cases when tRNA fragmentation was already evident in exponentially growing cells, the condition with the apparent weakest signal was taken as 1.0. The strongest fragmentation efficiency for each tRNA species is highlighted in red. Mean and standard deviations of three biological replicates are shown. Maximal tRNA fragmentation efficiencies (compared to the full-length tRNA signals) for each tRNA species are given in the last column (blue). Only five tRNA species (Arg, Asn, His, Met i, Sel) did not show any trace of tRNA fragmentation. Non detectable tRNA fragmentation is indicated in grey.

a

b

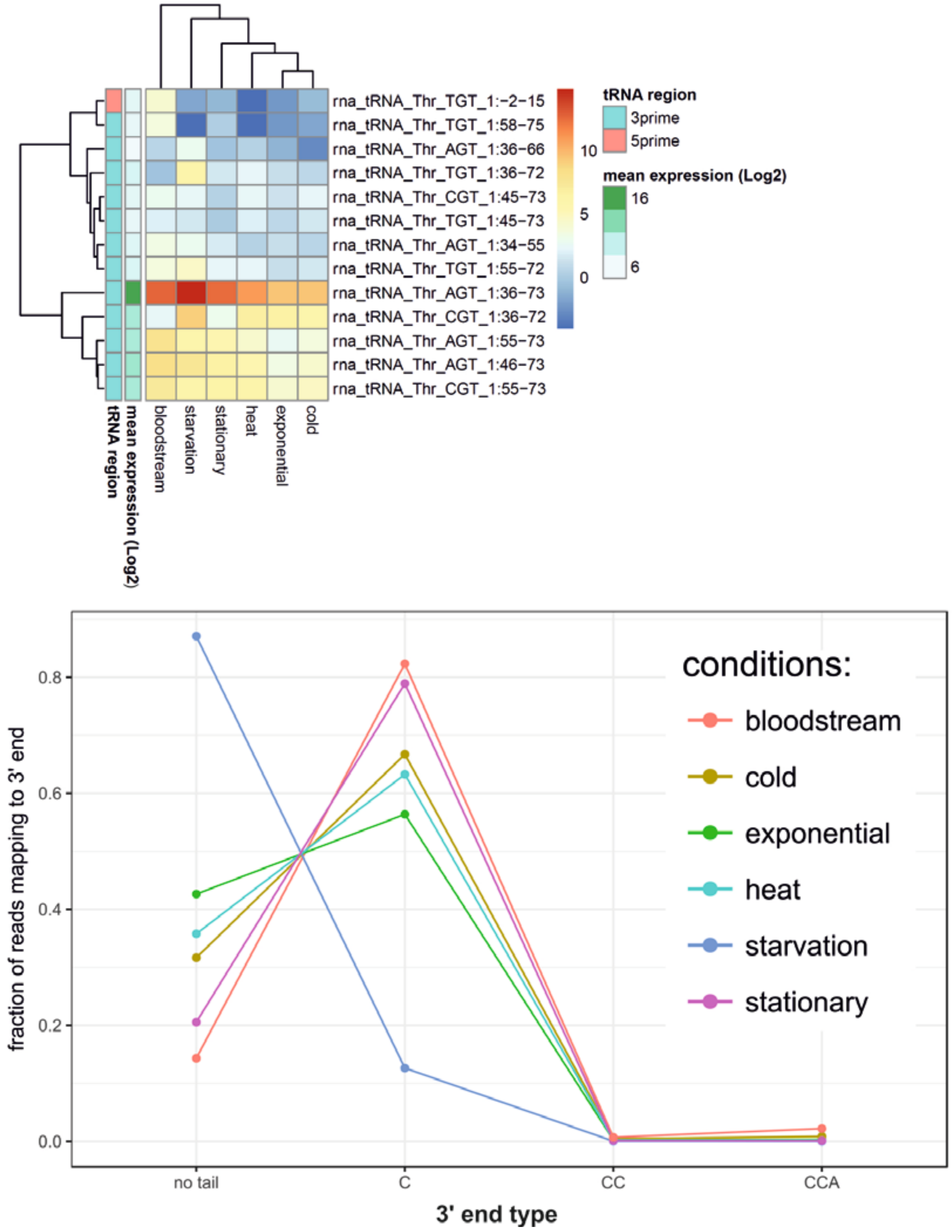

Supplementary Figure 2: Bioinformatics analyses of tRNA^{Thr}-derived reads obtained in different *T. brucei* libraries. **a** Heat map showing the abundance of the tRNA^{Thr}-derived fragments. The color scale represent the normalized expression (cpm – counts per million) shown in Log_2 scale. The mean expression level of the various tRNA-derived fragments is depicted in the green column. All but one tRNA^{Thr}-derived fragments originate from the 3' part of tRNA^{Thr} molecule (left column labelled 'tRNA region'). **b** All reads originating from tRNA^{Thr} (isoacceptor with AGU anticodon) that mapped to the 3' end of the gene, were analysed to determine the 3'-tail identities. For each condition, the fraction of reads without 3'-tail, with C, CC or CCA tails were estimated.

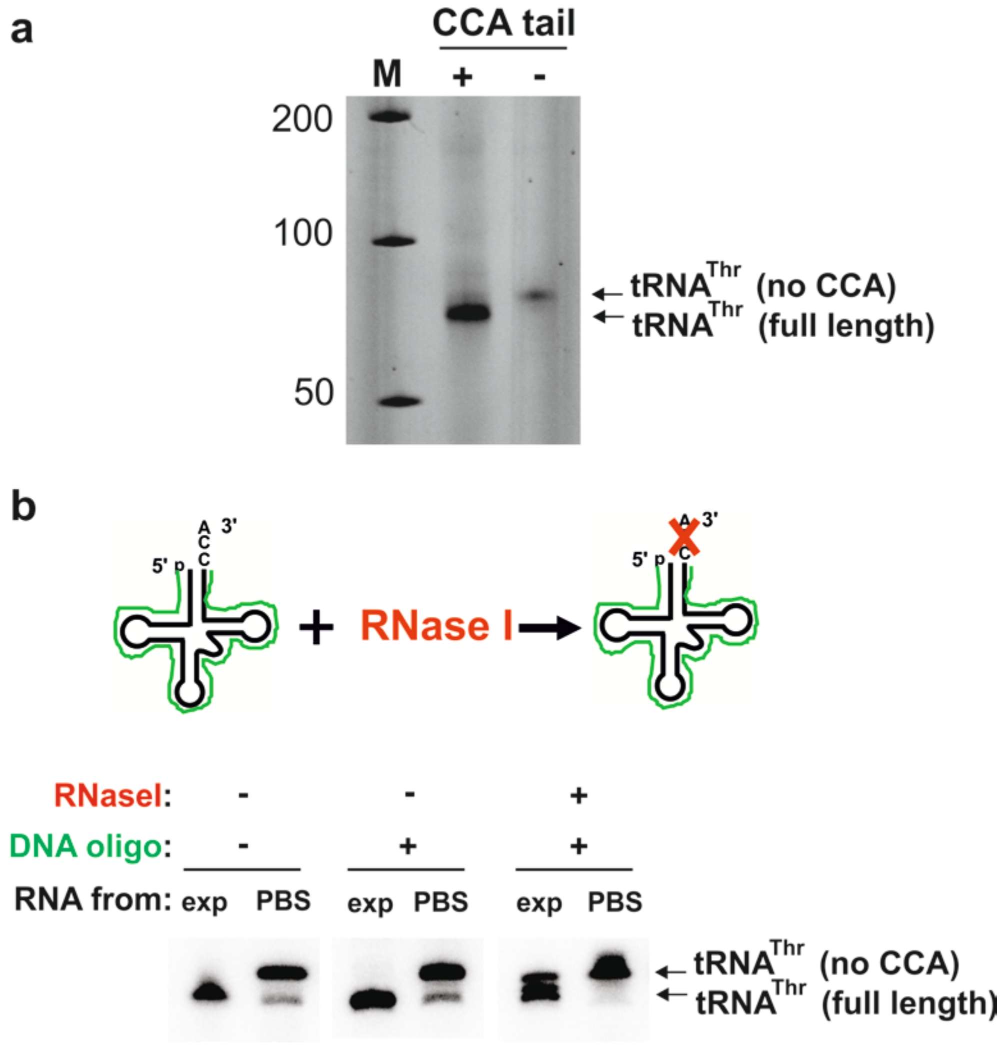

Supplementary Figure 3: The removal of the tRNA 3'-CCA tail affects gel electrophoresis. **a**, **b** $tRNA^{Thr}$ lacking the 3'-CCA runs more slowly during denaturing PAGE. (a) Denaturing polyacrylamide gel electrophoresis of *in vitro* transcribed *T. brucei* $tRNA^{Thr}$ containing (+) or lacking (-) the 3'-CCA tail. M denotes the FastRuler ultra low range DNA ladder (*Thermo*). Bands were visualized by EtBr staining. (b) Total *T. brucei* RNA was prepared either from exponentially growing cells (exp) or from cells starved for 2 h in PBS. Subsequently a DNA oligonucleotide (green) complementary to the entire $tRNA^{Thr}$ sequence except the 3' CCA tail was annealed and RNAse I was added and incubated at room temperature for 30 minutes. This treatment removes only the unprotected single stranded 3'-CCA overhang. After PCI extraction and denaturing gel electrophoresis, northern blot analysis with a radiolabelled DNA probe against $tRNA^{Thr}$ was performed.

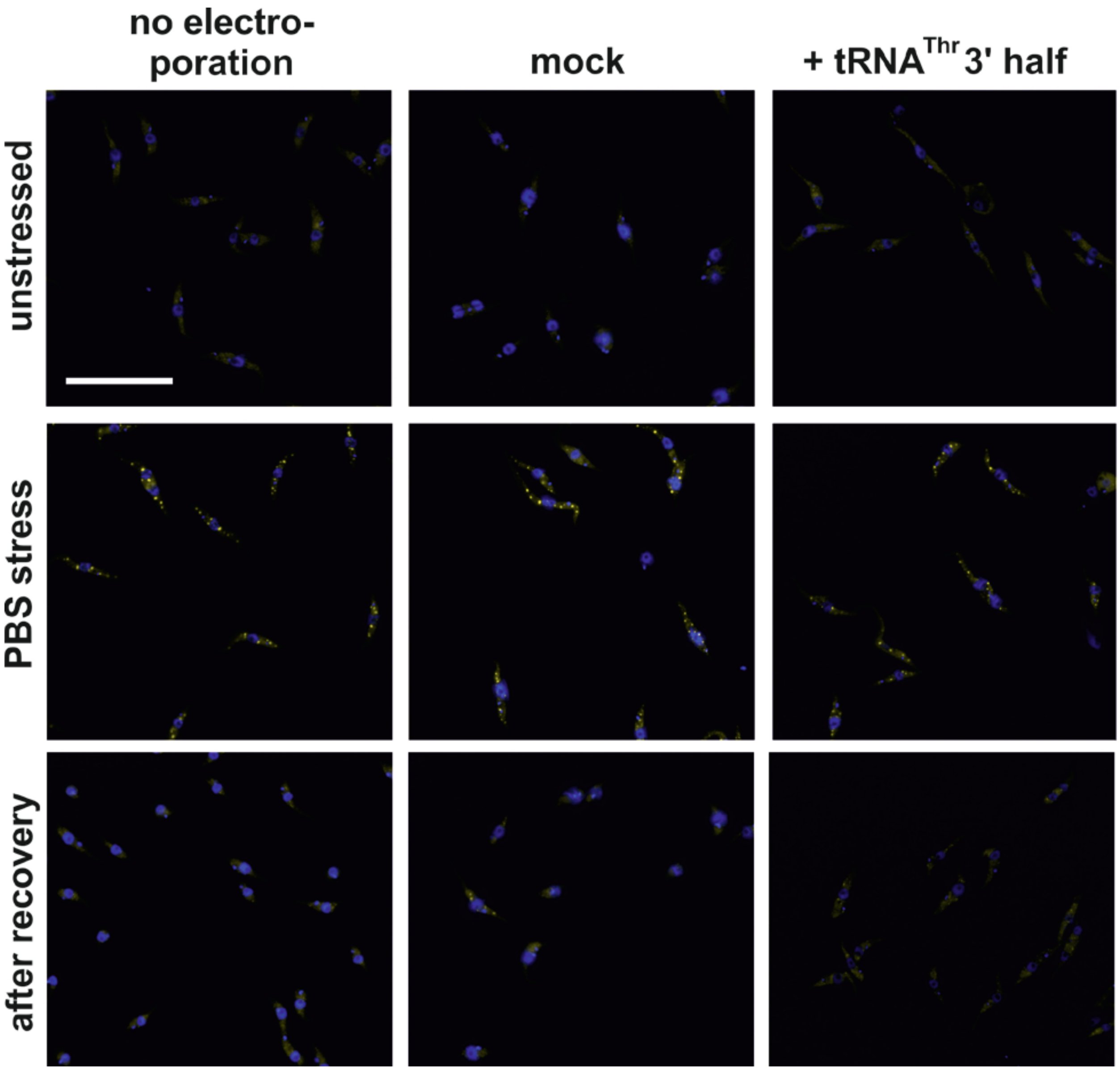

Supplementary Figure 4: Stress granule formation and clearance is independent of tRNA^{Thr} 3'-half abundance. In order to test if the tRNA^{Thr} 3' half has an effect on stress granule (SG) assembly or disassembly during the recovery phase we used a *T. brucei* cell line expressing DHH1 N-terminally tagged (eYFP) as a granule marker. Representative fluorescence images of cells after two hours starvation (PBS stress) followed by a one hour recovery in full media are shown. Merged images were counterstained with DAPI (blue) to identify the nuclei and the kinetoplast. The SG number and SG disassembly is not affected by any of the tested conditions: no electroporation (left column), electroporation in the absence (mock; middle column) or presence of tRNA^{Thr} 3' halves (right column). Scale bar: 25 µm

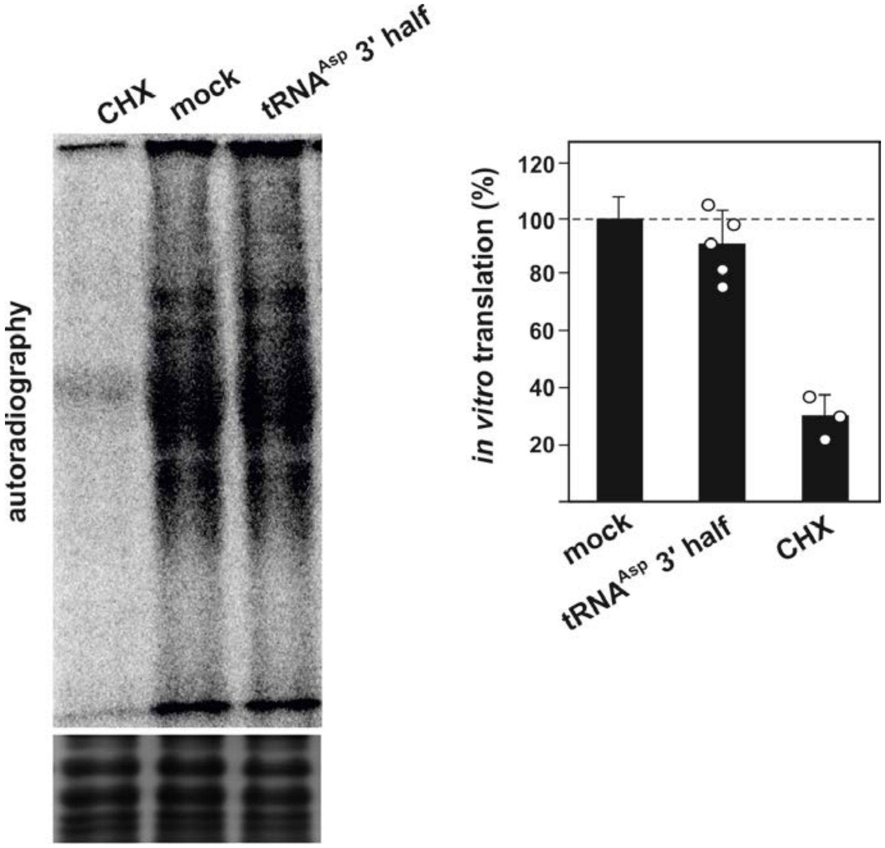

Supplementary Figure 5: The *T. brucei* tRNA^{Asp} 3' half has no effect on *in vitro* translation. The ^{35}S methionine incorporation during *T. brucei in* vitro translation was monitored in the absence (mock) or presence of 500 pmol tRNA^{Asp} 3' half. Cycloheximide (CHX) served as translation inhibition control. The Coomassie stained protein gel serves as loading control and is shown below the autoradiogram of the SDS polyacrylamide gel. Quantification (mean and standard deviations) of three to five independent *in vitro* translation reactions is shown on the right.

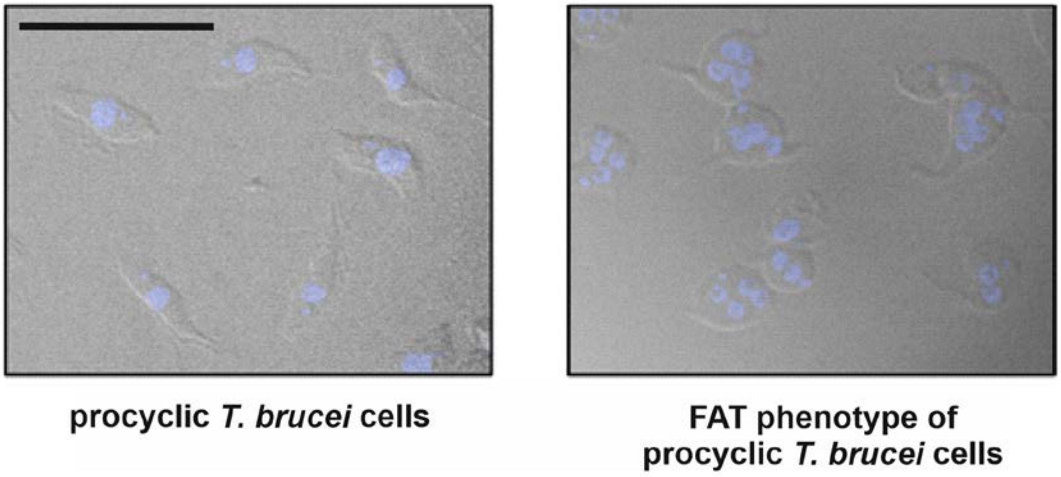

Supplementary Figure 6: FAT phenotype of procyclic *T. brucei* cells. siRNA-315 targeting the α-tubulin mRNA was used to test the electroporation efficiency of short RNAs into procyclic *T. brucei* cells. Successful introduction of the siRNA-315 was revealed by the observation of FAT cells containing multiple nuclei 18 hours after electroporation. Scale bar: 25 µm

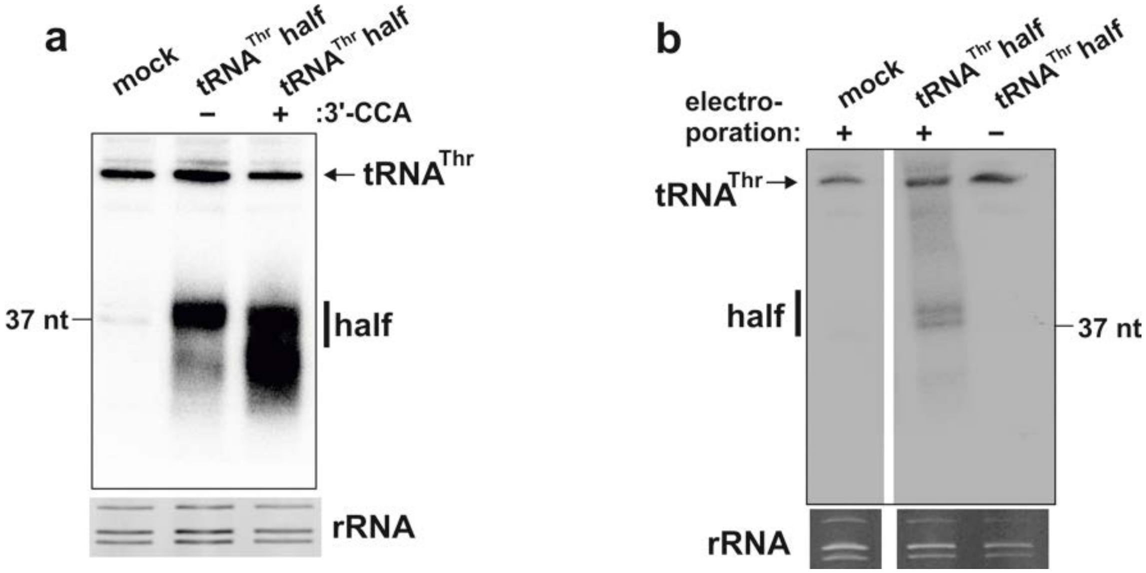

Supplementary Fig. 7: Electroporation of $tRNA^{Thr}$ 3' halves into *T. brucei*. **a** Successful electroporation of *in vitro* transcribed $tRNA^{Thr}$ 3' halves into *T. brucei* cells was monitored by northern blot analysis. Total RNA extracted from cells electroporated in the absence of any added nucleic acids (mock) or with the $tRNA^{Thr}$ 3' half (-/+ 3'-CCA tail) was used for northern blot analysis. **b** In the absence of electroporation no *in vitro* transcribed $tRNA^{Thr}$ 3' half molecules can be detected by northern blot analysis. The experiment was performed as in (a) but also using cells that were not electroporated in the presence of synthetic $tRNA^{Thr}$ 3' halves as a control.

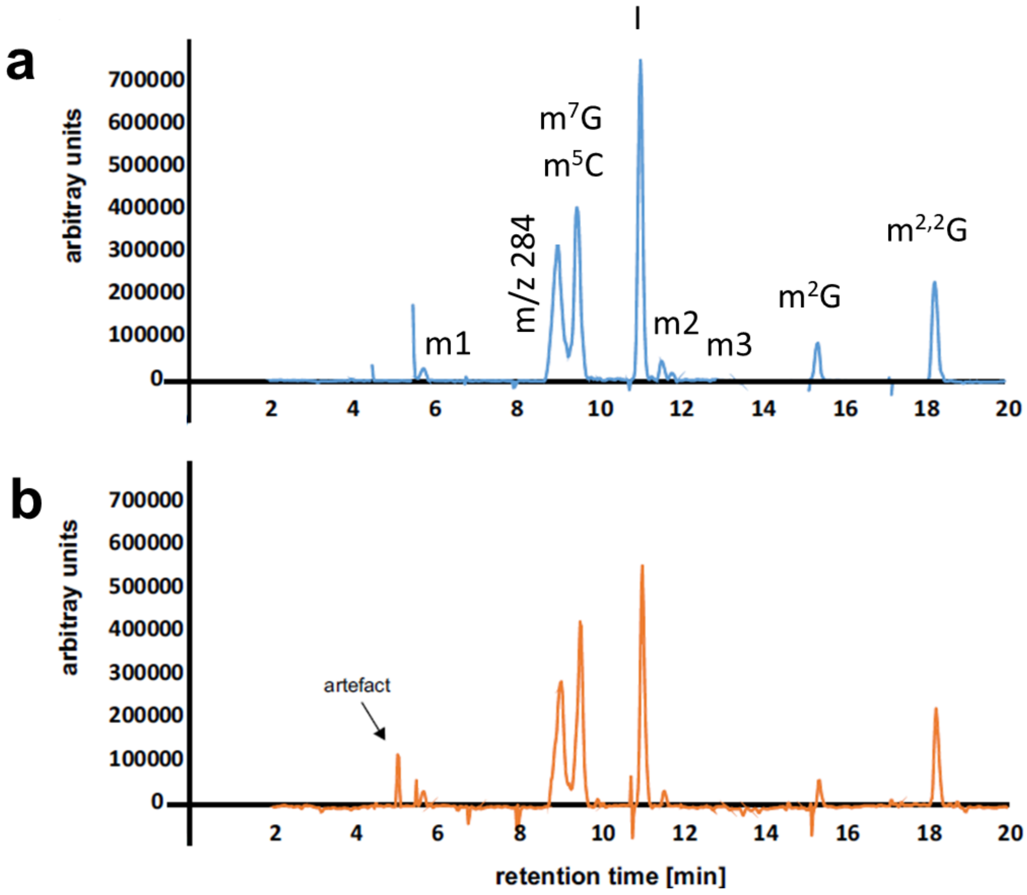

Supplementary Figure 8: tRNA^{Thr} modifications do not change during starvation stress as assessed by LC-MS/MS analysis. Affinity purified tRNA^{Thr} isolated from exponentially growing *T. brucei* cells (**a**) or starved cells (**b**) were digested to mononucleosides, separated by HPLC and analyzed by an NLS approach aimed at detecting nucleosides containing a ribose or methylated ribose. Major signals were obtained for the five known modifications m^7G, m^5C, I, m^2G and $m^{2,2}G$. One major signal of m/z 284 (retention time ~9 minutes) could not be plausibly matched to known modifications[1]. Three minor signals m1-m3 were detected, of which two were attributed to m^3C (m1) and m^3U (m3), likely arising from minor contaminations of the RNA preparations. One sharp peak with a retention time near 6 minutes was identified as an HPLC-based artefact that also occurred in blank runs. Signals in panels (a) and (b) are shown on arbitrary scales as detailed in the supplementary methods section. A comparative analysis of tRNA^{Thr} from cells exposed to normal (panel a) or starvation conditions (PBS, panel b) did not show any significant differences for any of the signals.

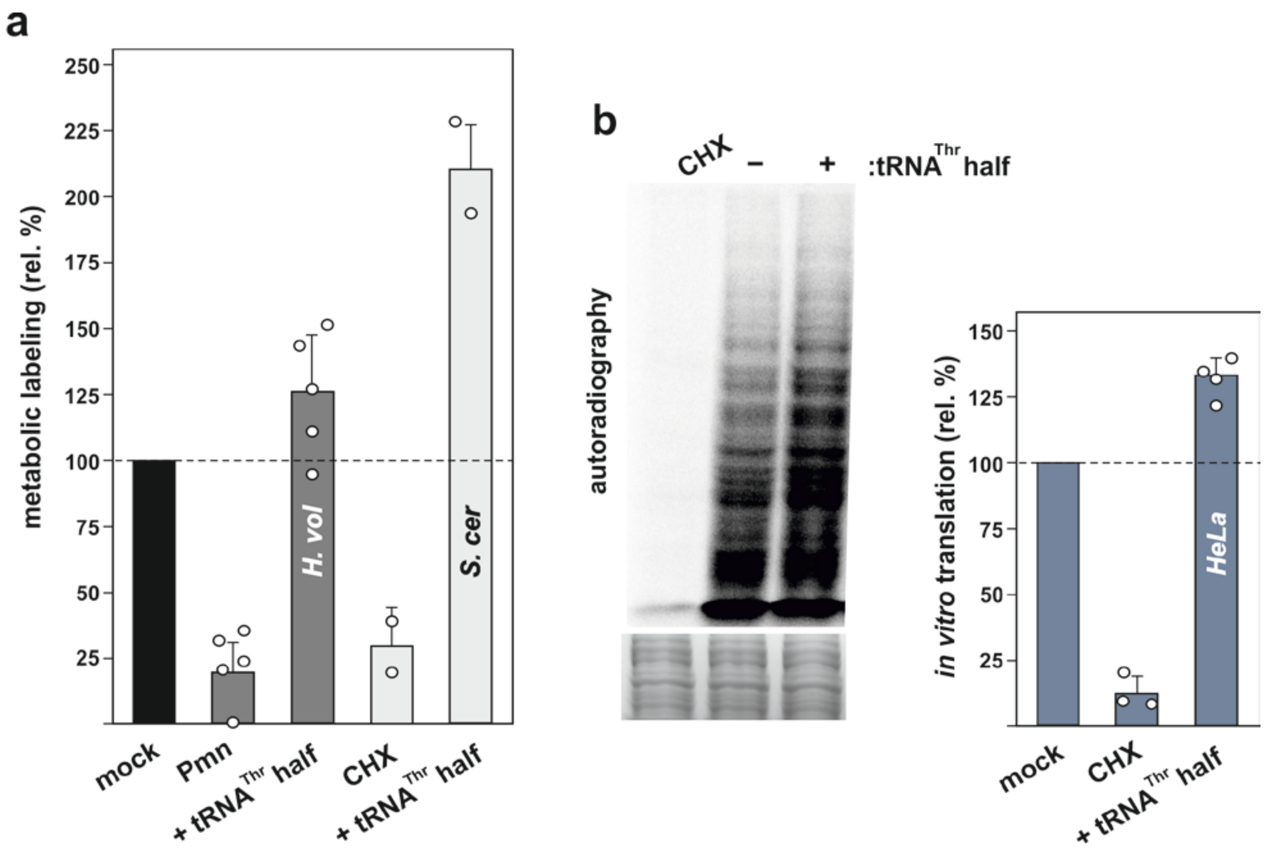

Supplementary Figure 9: The *T. brucei* $tRNA^{Thr}$ 3' half also stimulates translation in other species. **a** The $tRNA^{Thr}$ half stimulates metabolic labelling in the halophilic archaeon *H. volcanii* (grey bars; n = 5) and in the yeast *S. cerevisiae* (light grey bars: n = 2). The antibiotics puromycin (Pmn) and cycloheximide (CHX) served as translation inhibitor controls. **b** Addition of the *T. brucei* $tRNA^{Thr}$ half into HeLa cell-based *in vitro* translation reactions. On the left, the autoradiograph of a representative SDS polyacrylamide gel is shown. The Coomassie stained protein gel serves as loading control and is shown below the autoradiogram. Quantification of four independent *in vitro* translation experiments in the absence (mock) or presence of the $tRNA^{Thr}$ 3' half is shown on the right. Addition of the translation inhibitor cycloheximide (CHX) serves as the translation control for the assay (n = 3).

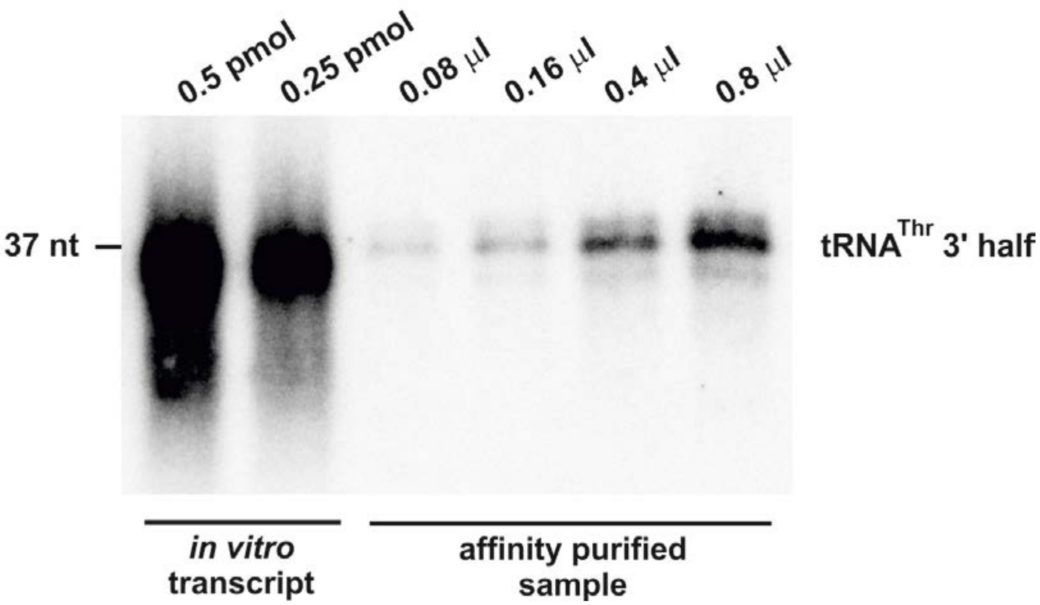

Supplementary Figure 10: Quantification of the amount of affinity purified endogenous *T. brucei* $tRNA^{Thr}$ 3' halves. After affinity purification using a biotinylated antisense oligonucleotide (see Methods of the main text) the $tRNA^{Thr}$ 3' halves were resuspended in 15 – 20 µl water and various aliquots (corresponding to 0.08 to 0.8 µl of the total affinity purified sample) were loaded on a denaturing polyacrylamide gel and used for subsequent northern blot analysis. To quantify the amount of the endogenous tRNA halves isolated, known amounts of *in vitro* transcribed $tRNA^{Thr}$ 3' halves of 37 nucleotides length were loaded alongside. Based on this approach the total amount of affinity purified $tRNA^{Thr}$ 3' halves was ~2 pmol whereas ~0.8 – 1 pmol thereof was applied for the *in vitro* translation reactions shown in Fig. 6c of the main text.

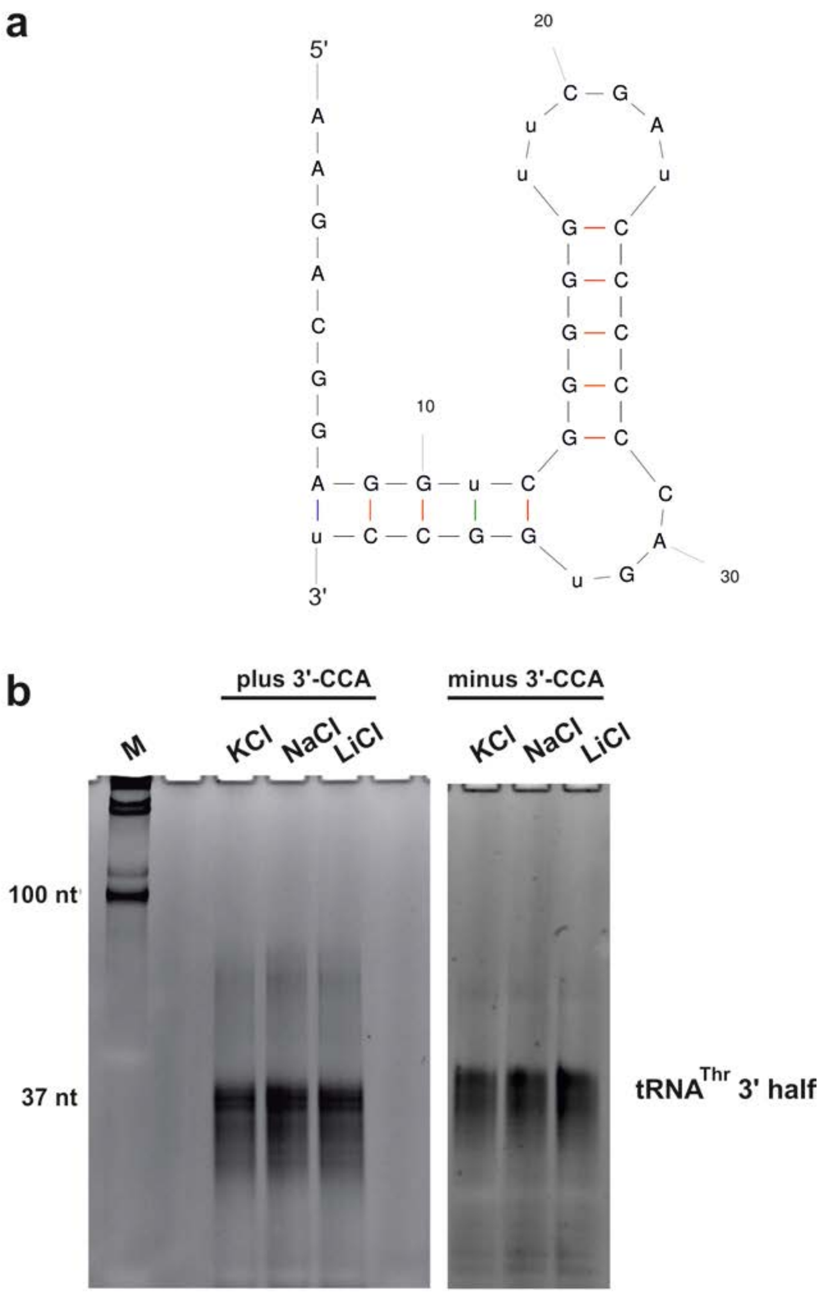

Supplementary Figure 11: The structure of the *T. brucei* $tRNA^{Thr}$ 3’ half. **a** The secondary structure of the $tRNA^{Thr}$ 3’ half was predicted by Mfold and the RNAfold web server (University of Vienna). **b** The $tRNA^{Thr}$ 3’ half (either with or without the 3’-CCA sequence) was incubated for 10 min at 95 °C in the presence of 100 mM of different monovalent ions known to stimulate (KCl, NaCl) or prevent (LiCl) RNA G-quadruplex formation (ref. number 35 of the main text). Subsequent the samples where incubated overnight at room temperature before running them on a 15% non-denaturing polyacrylamide gel. The gels were then stained with ethidium bromide. In none of the applied conditions do we see evidence for G-quadruplex formation. According to Lyons et al. 2017 (ref. number 35 of the main text) tRNA halves that adopt G-quadruplex conformation migrate between the 100 nt and 200 nt RNA size marker (M) range on the gel.

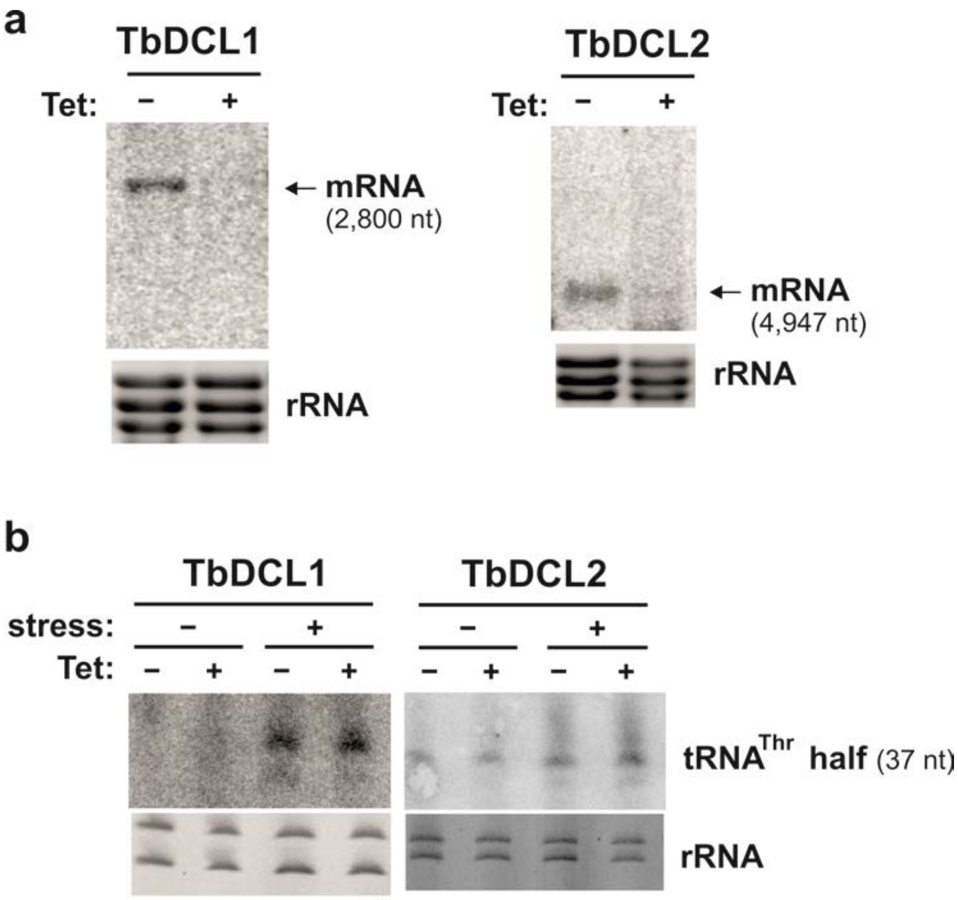

Supplementary Figure 12: shRNA-mediated knock-down of the dicer-like proteins TbDCL1 and TbDCL2 in *T. brucei*. a The expression of shRNAs directed against TbDCL1 (left) or TbDCL2 (right) was induced by tetracycline for three days. Northern blot analyses demonstrate efficient knock-down of both mRNAs. **b** Northern blot analyses was used to monitor the $tRNA^{Thr}$ 3' half before and after shRNA expression (Tet - and Tet +, respectively). It is obvious that the appearance of the $tRNA^{Thr}$ 3' half upon starvation stress (2 h in PBS) is independent of the TbDCL1 or TbDCL2 knock-down. In all cases EtBr stained rRNAs serve as loading controls.

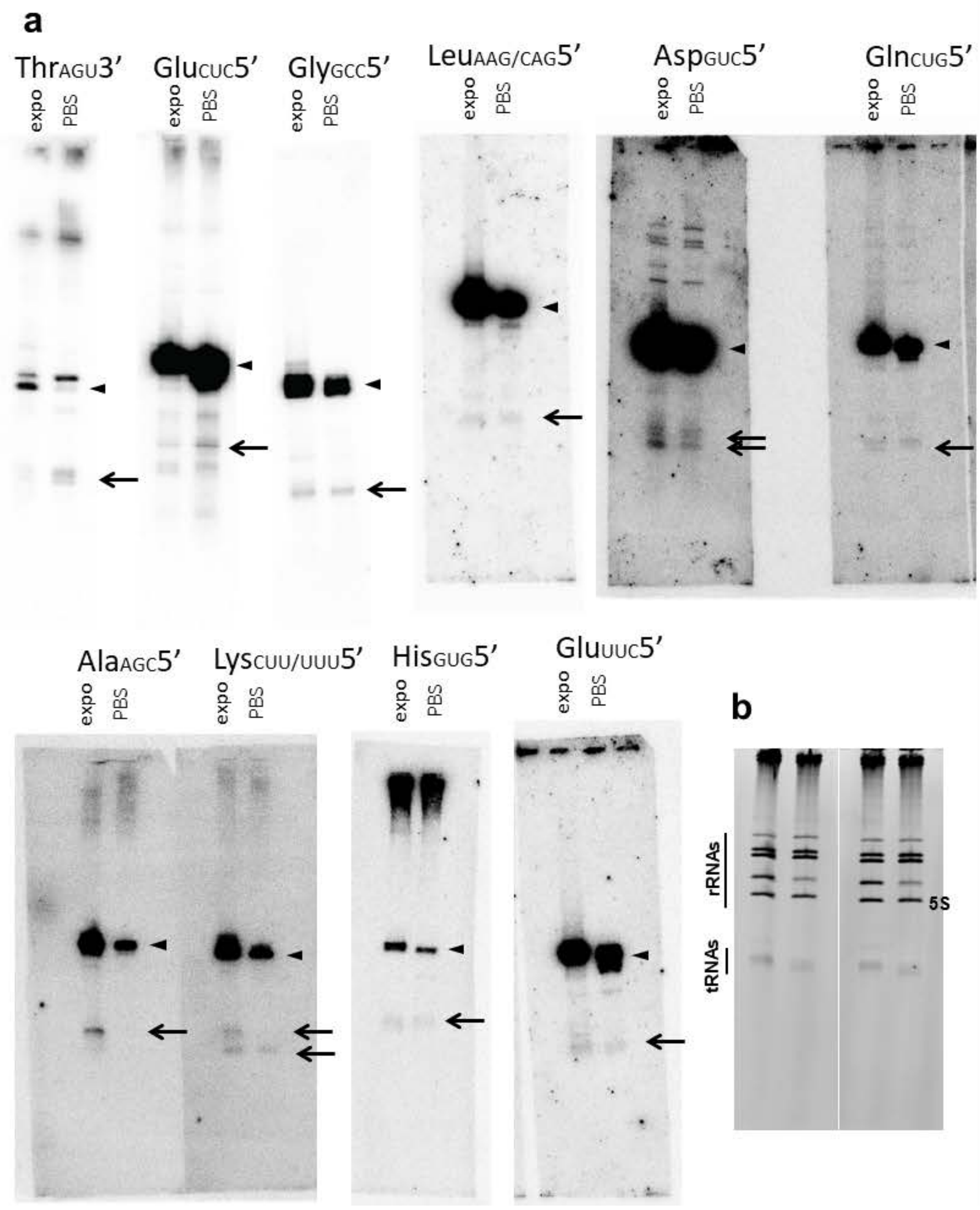

Supplementary Figure 13: Uncropped northern blots and gels related to Figure 1d of the main text. **a** Uncropped northern blot scans of tRNA-derived fragments (arrows) and full length tRNAs (arrow heads). **b** Full length ethidium bromide stained gels used as loading controls. The location of rRNAs (and the 5S rRNA) as well as bulk tRNAs is indicated.

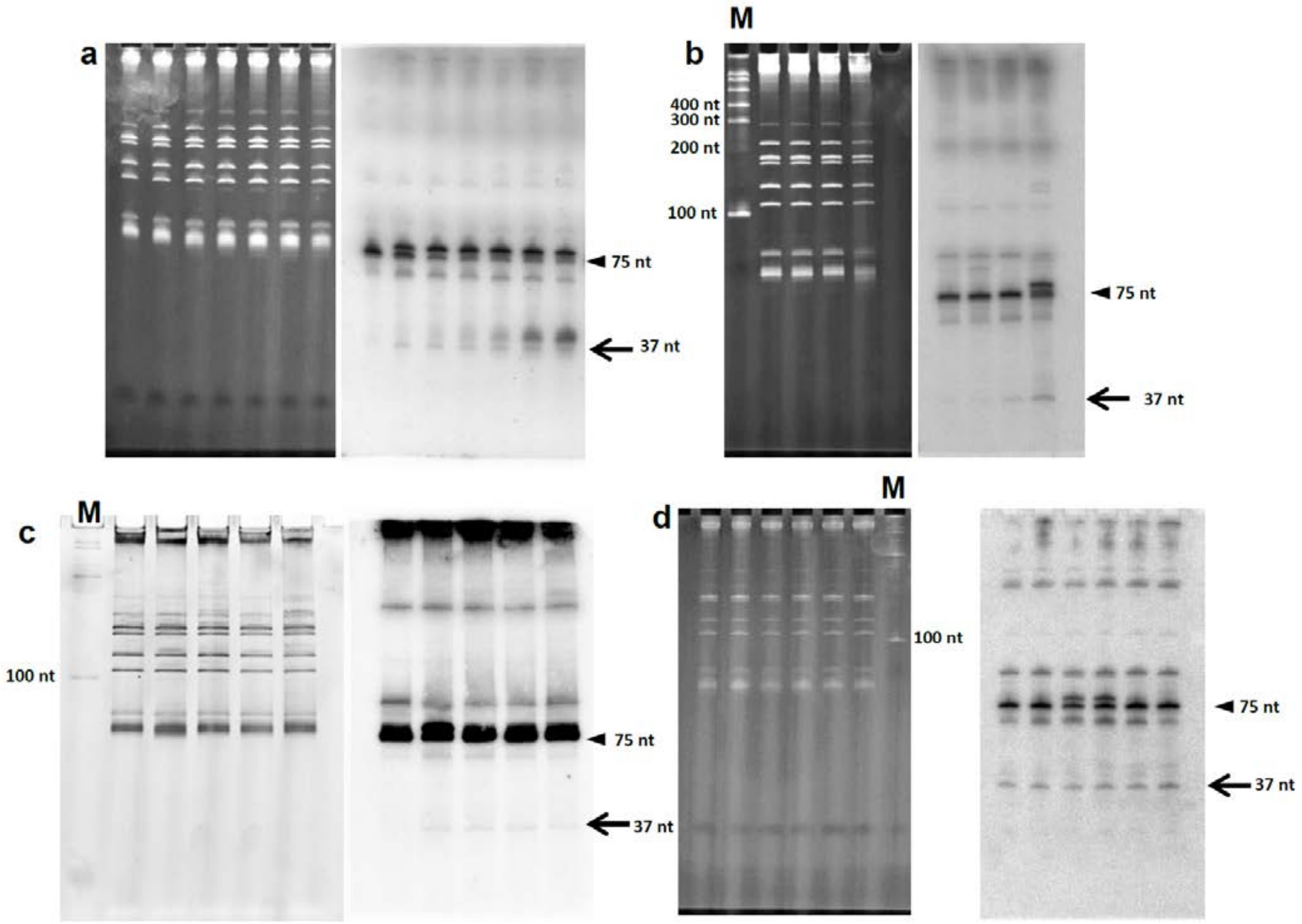

Supplementary Figure 14: Uncropped northern blots and gels related to Figure 2 of the main text. **a-d** Full length ethidium bromide stained gel (left) used as loading controls. M depicts the RiboRuler Low Range RNA ladder (*Thermo Scientific*). Uncropped northern blot scans (right) of the $tRNA^{Thr}$ 3' half (arrow; 37 nucleotides) and full length $tRNA^{Thr}$ (arrow head; 75 nucleotides). (a) Data for Figure 2a; (b) Data for Figure 2b; (c) Data for Figure 2c; (d) Data for Figure 2d.

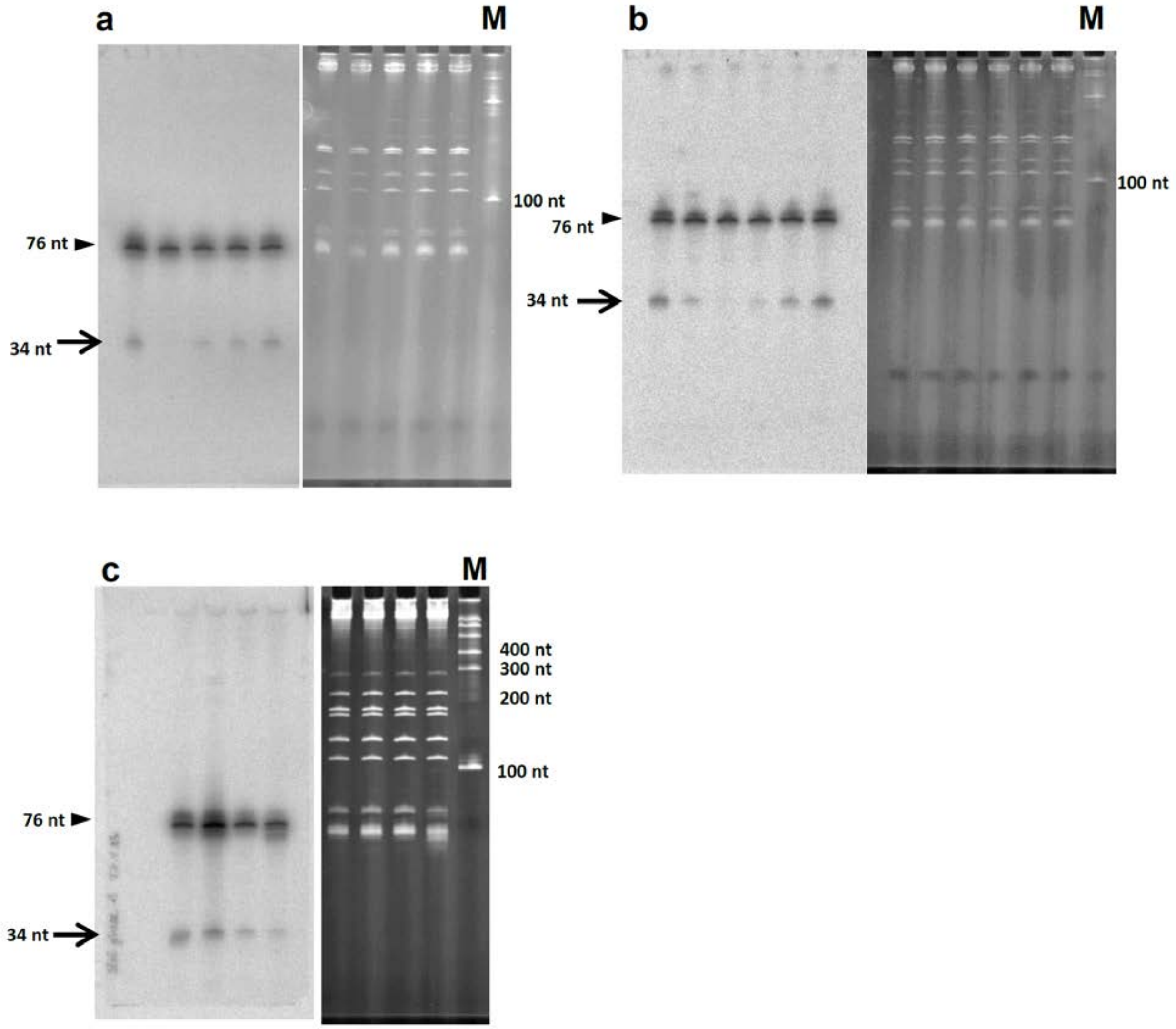

Supplementary Figure 15: Uncropped northern blots and gels related to Figure 3 of the main text. a-c Full length ethidium bromide stained gel (right) used as loading controls. M depicts the RiboRuler Low Range RNA ladder (*Thermo Scientific*). Uncropped northern blot scans (left) of the $tRNA^{Ala}$ 5' half (arrow; 34 nucleotides) and full length $tRNA^{Ala}$ (arrow head; 76 nucleotides long). (a) Data for Figure 3a; (b) Data for Figure 3b; (c) Data for Figure 3c.

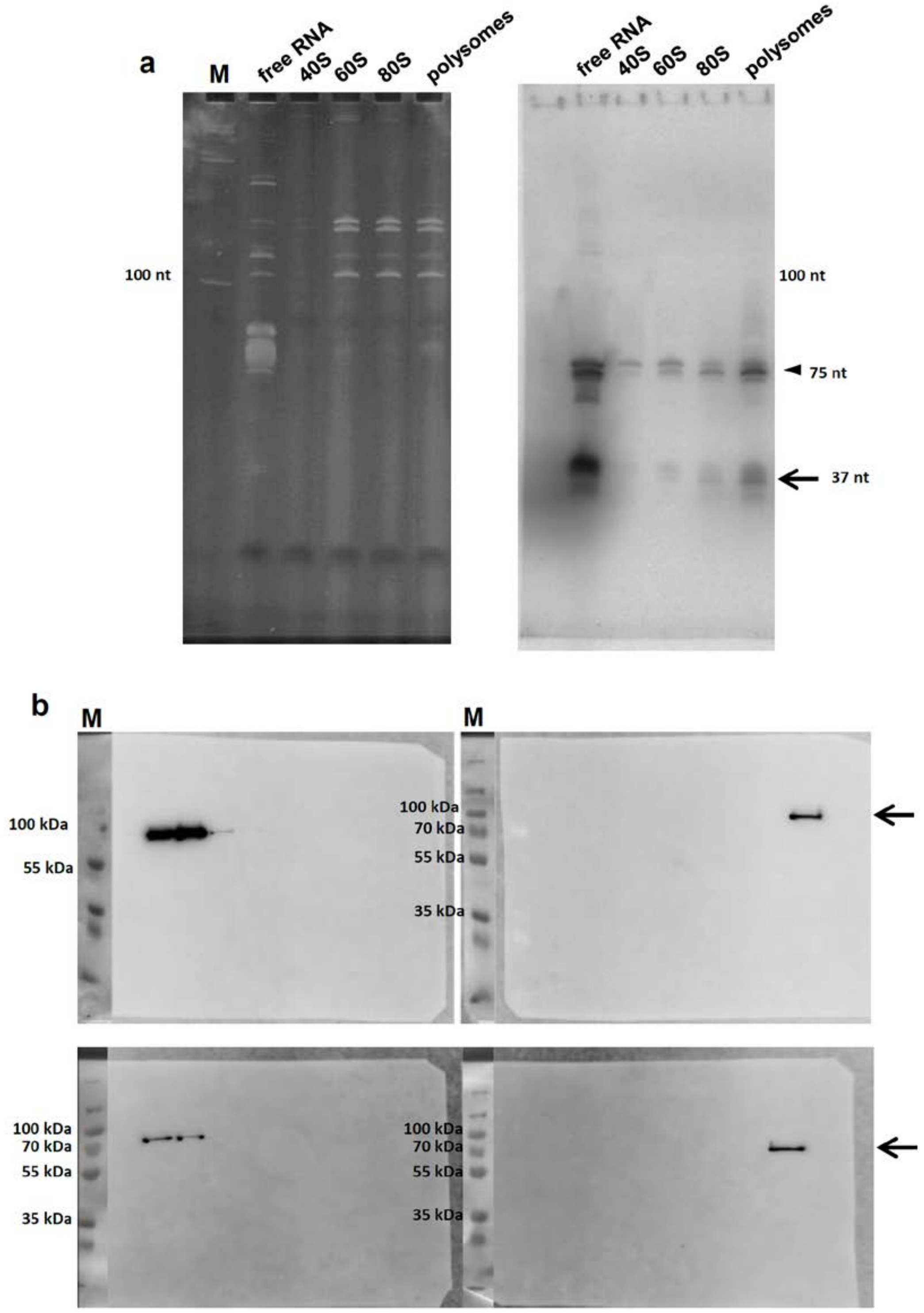

Supplementary Figure 16: Uncropped blots and gels related to Figure 4 of the main text. **a** Full length ethidium bromide stained gel (left). M depicts the RiboRuler Low Range RNA ladder (*Thermo Scientific*). Uncropped northern blot scan (left) of the $tRNA^{Thr}$ 3' half (arrow; 37 nucleotides) and full length $tRNA^{Thr}$ (arrow head; 75 nucleotides). **b** Uncropped western blot to detect DHH1-YFP (arrow). M depicts the PageRuler pre-stained protein ladder (*Thermo Scientific*). (a) Data for Figure 4b; (b) Data for Figure 4c.

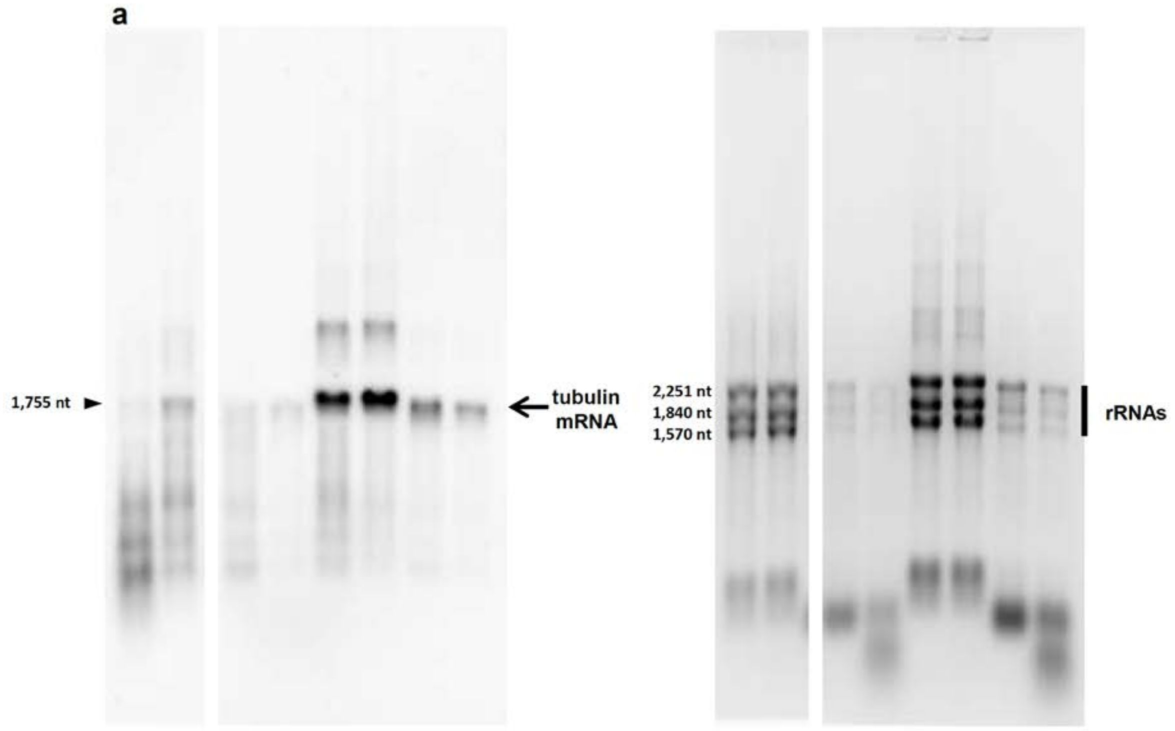

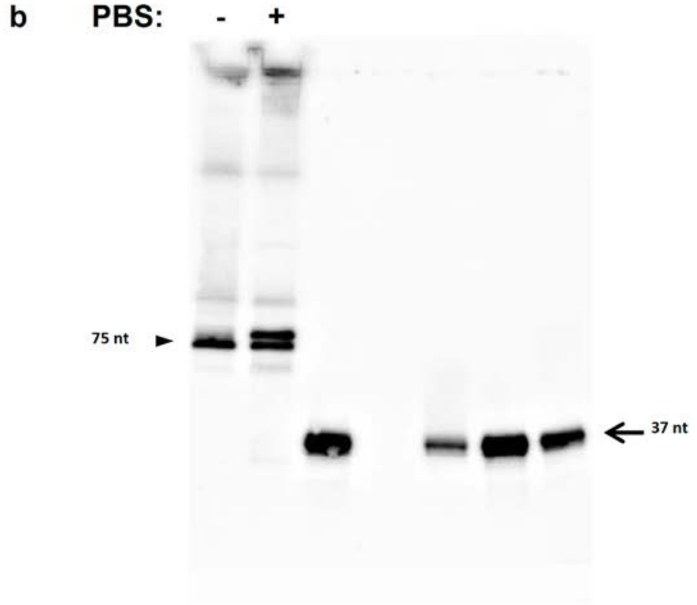

Supplementary Figure 17: Uncropped blots and gels related to Figure 5 and 6 of the main text. **a** Uncropped northern blot (left) to detect tubulin mRNA (arrow). Full length ethidium bromide stained gel (right) was used as loading control. The location and sizes of rRNAs is indicated. **b** Uncropped northern blot for detecting affinity-purified endogenous $tRNA^{Thr}$ 3' halves (arrow). Total RNA from exponentially growing *T. brucei* or cells stressed for two hours in PBS (+) was used as marker. The position of full length $tRNA^{Thr}$ is labelled with an arrow head. (a) Data shown refer to Figure 5d; (b) Data shown refer to Figure 6b.

SUPPLEMENTARY METHODS

Fluorescence microscopy

Stress granules (SG) were monitored by microscopy in unstressed and starved (PBS stress) *T. brucei* cells expressing an endogenous copy of DHH1 N-terminally tagged (eYFP) as a granule marker (strain was constructed according to the constructs from M. Carrington & S. Kramer). $3x10^6$ cells were washed in 1x ice cold PBS and resuspended in 200 µl of 1x PBS. 100 µl was placed onto a cover slides (10 mm) and incubated for 10 minutes to let the cells settle. The PBS was removed and the cells were fixed with 150 µl ice cold methanol for 10 minutes at -20°C. After removal of the methanol the slide was washed three times with 150 µl 1x PBS with a three minute incubation time. After drying, the slides were mounted with a drop of DNA-staining agent VECTASHIELD (*Reactolab*) that labels both the nuclei and the kinetoplast DNA network. Images were acquired with a DFC360 FX monochrome camera (*Leica Microsystems*) and a DMI600B microscope (*Leica Microsystems*). Image analysis was done using LAS X software (*Leica Microsystems*) and Adobe Photoshop CS5.1 (Adobe).

In vitro translation using HeLa cells

6 µl total HeLa cell lysate was pre-incubated in the presence of 500 pmol $tRNA^{Thr}$ 3' halves for 10 min at 35°C. Then the translation mix was added (to a final concentrations of 0.02 M creatine phosphate, 1.4 mg/ml yeast bulk tRNA, 0.1 mg/ml creatine phosphokinase, 0.625 µl L-^{35}S-methionine (10 µCi/µl) and translational cocktail as described for *T. brucei*) to a final volume of 12 µl and incubated at 35°C for further 30 min. Finally the reaction was stopped by the addition of 4x Laemmli buffer, the proteins separated by SDS-PAGE and methionine incorporation assessed by phosphorimaging.

Affinity purification of full-length tRNA^{Thr} and LC-MS/MS analysis

Affinity purification of tRNA^{Thr} from total RNA extracted from unstressed or PBS stressed *T. brucei* was performed as described in the main text for the tRNA^{Thr} 3' half using size-selected total RNA (30 – 40 nt) as input material. The affinity purified and DNase-treated tRNA^{Thr} was then loaded onto an 8 % polyacrylamide gel. The tRNA bands were excised from the gel, the RNAs passively eluted into 0.3 M NaOAc and 1 mM EDTA and subsequently purified via PCI extraction followed by ethanol precipitation. Subsequently RNA was hydrolyzed to nucleosides using nuclease P1 (*Roche Diagnostics*, Germany), and snake venom phosphodiesterase (*Worthington*, USA) as described[2]. Nucleosides were separated on a Synergy Fusion RP18 column (4 µm particle size, 80 Å pore size, 250 mm length, 2 mm inner diameter) from *Phenomenex* (Germany). An Agilent 1260 series HPLC tower equipped with a diode array detector (DAD) and a Triple Quadrupole mass spectrometer (*Agilent* 6460) was used for mass spectrometric analysis (details in ref. 2). For comparison the samples mass signals were normalized to the amount of canonical nucleosides, as obtained from the UV traces, hence the scales on the Y-axes shown in Supplementary Figure 8 represent arbitrary units.

Northern blot analyses

For northern analyses monitoring all tRNA isoacceptors in *T. brucei* (either targeting the 5' or 3' halves) the following 5' end-labeled DNA oligonucleotides were used:

Ala (AGC): 5'-GGCGCTCTACCATCTGAGCTACA-3'; Ala (AGC): 5'-TATCGATCCCAATACCTCCCGCA -3'; Arg (ACG): 5'-GATGCTCTTCCATTGAGCCAC-3'; Arg (ACG): 5'-GACAGGATTCGAACCTGCAACC-3'; Asn (GUU): 5'-CCCGTTGGATTCGAACCAACGACC-3'; Asn (GUU): 5'-GAATTGAACCCGGGTCACCCGCG-3'; Asp (GUC): 5'-ACTAACCACTATACTACCGAG-3'; Asp (GUC): 5'-GAATTGAACCCGGGTCACCCGCG-3'; Cys (GCA): 5'-TGCTCTACCACTGAGCTATGAATC-3'; Cys (GCA): 5'-CACCCGGGTTTGAACCGGGGAC-3'; Glu (CUC): 5'-

GTTCTAGCCACTATACCACAC-3'; Glu (UUC): 5'-AGCCAGATGTTCTAACCGTT-3'; Glu (CUC/UCC): 5'-AGACCCGGGTTCGATCCCCGG-3'; Gln (CUG): 5'-TAACCGCTACACTATGGGAGC-3'; Gln (CUG): 5'-GGACTCGAACCAGGGTTATCGGAT-3'; Gly (GCC): 5'-CTACCACTAGACCAACTGCGC-3'; Gly (UCC): 5'-CTTACCGTTGGACCACGATTGC-3'; Gly (GCC/UCC): 5'-CCGGGAGTCGAACCCGGGTC-3'; His (GUG): 5'-TGTTCTGCCACTGAACTATCTTC-3'; His (GUG): 5'-AACGTGAGGGAAGACCGGGAATCGA-3', Ile (AAU): 5'-CTAACCGACTGAGCTATAGGAGC-3'; Ile (AAU): 5'-TGCTCCCAACAGGGGTCGAACCTGTGA-3'; Leu (AAG/CAG): 5'-CGTCTTAGACCACTCGACCAT-3'; Leu (CAA/UAG/CAG/AAG): 5'-GTGGGGTTTGAACCCACGCC -3'; Lys (CUU/UUU): 5'-CGTGCGCTCTACCGACTGAGCTAG-3'; Lys (CUU/UUU): 5'-GGGATCGAACCCACGACCACACG-3'; Met (CAU): 5'-GCGCTGCCGACTGCGCCACGCTCGC-3'; Met (CAU): 5'-GCTCGAACTCACGACCTTGGGA-3'; Met in (CAU): 5'-GCGCTTCCCCTGCGCCACGGTGC-3'; Met in (CAU): 5'-GGTTTCGATCCAACGTCCTATGGG -3'; Phe (GAA): 5'-CTCTCCCAACTGAGCTATCGCGGC-3'; Phe (GAA): 5'-GCCGCGACCCGGGATCGAACCAG-3'; Pro (CGG/AGG): 5'-GAATCATGCCACTAGACCA-3'; Pro (CGG/AGG): 5'-CGGGAATTGAACCCGGGACCTC-3'; Sel (UCA): 5'-ACCCAGCACCAGCTGAGCTCATCG-3'; Sel (UCA): 5'-CACCACAAAGCCCGAATCGAAC-3'; Ser (AGA/CGA/UGA): 5'-CCCCGTAACCACTTGGGTATG-3'; Ser (AGA/CGA/UGA/GCU): 5'-TTCGAACCTGCGCGCGAGATC-3'; Thr (AGU): 5'-TCTGCCATTGAGCTAAGCGGCC-3'; Thr (AGU): 5'-ATCGAACCCCCGACCTCCGTCTT-3'; Trp (CCA): 5'-CCAATGCTCTACCACTGAGC-3'; Trp (CCA): 5'-GGGATTGAACCTGCGACCCCTGG-3'; Tyr (GUA): 5'-CTACCAATTGAGCTACAGAAGG-3'; Tyr (GUA): 5'-CCAGCGACCCTGTGATACCCGC-3'; Val (CAC): 5'-CCTAACCACTAGACGACCATCGC-3'; Val (AAC): 5'-CATAACCACCTAGACCATCAGCGC -3'; Val (UAC): 5'-CGGGGTTCGAACCCGCGACGTTC -3'; Val (AAC): 5'-CTCGAACCCGAGACCTTCGCCG -3'

SUPPLEMENTARY REFERENCES

1 Boccaletto, P. *et al.* MODOMICS: a database of RNA modification pathways. 2017 update. *Nucleic Acids Res* **46**, D303-D307, doi:10.1093/nar/gkx1030 (2018).

2 Thuring, K., Schmid, K., Keller, P. & Helm, M. Analysis of RNA modifications by liquid chromatography-tandem mass spectrometry. *Methods (San Diego, Calif* **107**, 48-56, doi:10.1016/j.ymeth.2016.03.019 (2016).
