# Supplementary material for: A tRNA half modulates translation as stress response in Trypanosoma brucei
Source: Nat Commun. 2019 Jan 10;10:118. doi: 10.1038/s41467-018-07949-6 (PMC6328589; doi:10.1038/s41467-018-07949-6)
Supplement: Supplementary file 3 — Description of Additional Supplementary Files [file 41467_2018_7949_MOESM3_ESM.pdf]

Description of Additional Supplementary Files

File Name: Supplementary Data 1

Description: Compilation of tRNA-derived sequencing reads. All identified tRNA reads obtained in the ribosome-associated ncRNA screen of T. brucei cells are listed. Information on the read length, location within the full-length tRNA, mean expression values and stress- and/or growth-specific abundance of each tRNA fragment is given.)
