# Supplementary material for: A tRNA half modulates translation as stress response in Trypanosoma brucei
Source: Nat Commun. 2019 Jan 10;10:118. doi: 10.1038/s41467-018-07949-6 (PMC6328589; doi:10.1038/s41467-018-07949-6)
Supplement: Supplementary file 5 — Reporting Summary [file 41467_2018_7949_MOESM5_ESM.pdf]

nature research

Corresponding author(s): Norbert Polacek, Marina Cristodero

Reporting Summary

Nature Research wishes to improve the reproducibility of the work that we publish. This form provides structure for consistency and transparency in reporting. For further information on Nature Research policies, see Authors & Referees and the Editorial Policy Checklist.

Statistical parameters

When statistical analyses are reported, confirm that the following items are present in the relevant location (e.g. figure legend, table legend, main text, or Methods section).

n/a	Confirmed	
☐	☒	The exact sample size (n) for each experimental group/condition, given as a discrete number and unit of measurement
☐	☒	An indication of whether measurements were taken from distinct samples or whether the same sample was measured repeatedly
☐	☒	The statistical test(s) used AND whether they are one- or two-sided<br>*Only common tests should be described solely by name; describe more complex techniques in the Methods section.*
☒	☐	A description of all covariates tested
☒	☐	A description of any assumptions or corrections, such as tests of normality and adjustment for multiple comparisons
☐	☒	A full description of the statistics including central tendency (e.g. means) or other basic estimates (e.g. regression coefficient) AND variation (e.g. standard deviation) or associated estimates of uncertainty (e.g. confidence intervals)
☐	☒	For null hypothesis testing, the test statistic (e.g. F, t, r) with confidence intervals, effect sizes, degrees of freedom and P value noted<br>*Give P values as exact values whenever suitable.*
☒	☐	For Bayesian analysis, information on the choice of priors and Markov chain Monte Carlo settings
☒	☐	For hierarchical and complex designs, identification of the appropriate level for tests and full reporting of outcomes
☐	☒	Estimates of effect sizes (e.g. Cohen's d, Pearson's r), indicating how they were calculated
☐	☒	Clearly defined error bars<br>*State explicitly what error bars represent (e.g. SD, SE, CI)*

Our web collection on statistics for biologists may be useful.

Software and code

Policy information about availability of computer code

Data collection	No previously unreported custom computer code or algorithm were used.
Data analysis	The deep sequencing data were analyzed using the open source APART pipeline, which was previously published by our group (Zywicki et al., Nucleic Acids Res. 40, 4013-4024, 2012)

For manuscripts utilizing custom algorithms or software that are central to the research but not yet described in published literature, software must be made available to editors/reviewers upon request. We strongly encourage code deposition in a community repository (e.g. GitHub). See the Nature Research guidelines for submitting code & software for further information.

Data

Policy information about availability of data

All manuscripts must include a data availability statement. This statement should provide the following information, where applicable:

- Accession codes, unique identifiers, or web links for publicly available datasets
- A list of figures that have associated raw data
- A description of any restrictions on data availability

All sequencing data generated in this study have been deposited at the European Nucleotide Archive (ENA) and can be accessed with the number PRJEB24915 [https://www.ebi.ac.uk/ena/data/view/PRJEB24915]. All other data are available from the corresponding authors on request.

Field-specific reporting

Please select the best fit for your research. If you are not sure, read the appropriate sections before making your selection.

☒ Life sciences ☐ Behavioural & social sciences ☐ Ecological, evolutionary & environmental sciences

For a reference copy of the document with all sections, see nature.com/authors/policies/ReportingSummary-flat.pdf

Life sciences study design

All studies must disclose on these points even when the disclosure is negative.

Sample size	There was no specific statistical method used to determine sample size. However, the experimental conditions were carried out in more than triplicate (biological replicates). Exception: The RNA Seq experiment was performed from cDNAs of ribosome-associated small RNAs isolated once but from several different growth conditions
Data exclusions	For northern blot analyses: RNA preparations that showed clear signs of degradations on ethidium bromide stained gels (e.g. smeary tRNA or rRNA bands) were excluded and not further analyzed.
Replication	All data shown could be replicated (note the n values).
Randomization	randomization was not relevant for this study
Blinding	Blinding was not relevant for this study. But the key experiments were performed by three independent researchers plus these three team performed similar experiments at different time periods and thus they did not physically overlap in the laboratory.

Reporting for specific materials, systems and methods

Materials & experimental systems

n/a	Involved in the study
☒	☐ Unique biological materials
☒	☐ Antibodies
☐	☒ Eukaryotic cell lines
☒	☐ Palaeontology
☒	☐ Animals and other organisms
☒	☐ Human research participants

Methods

n/a	Involved in the study
☒	☐ ChIP-seq
☒	☐ Flow cytometry
☒	☐ MRI-based neuroimaging

Eukaryotic cell lines

Policy information about cell lines

Cell line source(s)	Trypanosoma brucei procyclic stage 427, 29-13 or bloodstream forms New York single markers (NYSM) cell lines were used in this study. These cells were obtained from our collaboration partner and co-author A. Schneider.
Authentication	None of the cell lines used have been authentificated.
Mycoplasma contamination	T. brucei is not a host for Mycoplasma and thus not a source of possible contamination.
Commonly misidentified lines (See ICLAC register)	*Name any commonly misidentified cell lines used in the study and provide a rationale for their use.*
